# “It’s a lot of pain you’ve got to hide”: a qualitative study of the journey of fathers facing traumatic pregnancy and childbirth

**DOI:** 10.1186/s12884-022-04738-4

**Published:** 2022-05-24

**Authors:** A. Kothari, G. Bruxner, L. Callaway, J. M. Dulhunty

**Affiliations:** 1 Redcliffe Hospital, Anzac Avenue, Redcliffe, Queensland 4020 Australia; 2 The University of Queensland, St Lucia, Queensland 4067 Australia; 3 Metro North Mental Health Service, Butterfield St, Herston, Queensland 4029 Australia; 4 The Royal Brisbane and Women’s Hospital, Herston, Queensland 4006 Australia

## Abstract

**Background:**

This study aims to explore the emotional and behavioural responses and coping strategies of fathers or expectant fathers who faced a significant traumatic event during a partner's pregnancy, labour, or the postpartum period.

**Methods:**

This prospective qualitative study of 24 fathers was conducted at a public teaching hospital in Brisbane, Australia. ‘Traumatic pregnancy’ was defined as a pregnancy complicated by life-threatening or severe risk to the mother and the fetus, termination of pregnancy, intrauterine fetal death or stillbirth. Semi-structured interviews of participants were conducted 3-4 months after the traumatic event. An initial qualitative analysis with automatic coding was performed using Leximancer and later followed by a six-phase manual thematic analysis.

**Results:**

A pregnancy-related traumatic event had significant mental and physical impacts on fathers. Participants' reactions and coping strategies were varied and influenced by their background history, pre-existing vulnerabilities, and the gap between expectation and reality. Most fathers described a fluctuating state between their needs 'not being met' and 'being met'. These needs were conceptualised using Maslow’s hierarchy and Calman’s gap theory to construct a composite thematic model to depict the universal requirements of men facing a traumatic pregnancy or childbirth.

**Conclusions:**

A greater understanding of the needs of men and gaps in their care is urgently needed. A targeted effort is required to make maternity services father-inclusive. This approach may assist in preventing long term consequences on fathers, partners, and their children.

**Supplementary Information:**

The online version contains supplementary material available at 10.1186/s12884-022-04738-4.

## Introduction

Fathers play many important roles during the pregnancy and childbirth journey, including bystander, protector, supporter, gatherer and guardian of fact, and sometimes, decider or enforcer [1]. However, qualitative studies also suggest that fathers are often relegated to the role of an observer and feel vulnerable, excluded and dismissed [1, 2]. Some of the reasons why fathers experience these sentiments include sociocultural expectations to suppress their emotions and the fact that obstetric health care services around the world are still predominantly focused on the mother and baby [3]. Most perinatal research is on women’s experiences, possibly due to difficulties with recruiting men into social and psychological studies [4, 5]. Unfortunately, there is limited research available on first-hand accounts of fathers’ experiences of childbirth [3, 5–7].

The transition to fatherhood can be a difficult experience for fathers [8–11]. Even uncomplicated childbirth with good outcomes for mother and baby may be perceived as traumatic by men [6, 12]. The mental health impacts of pregnancy and childbirth on fathers may be exacerbated by the quality of maternity care, a sense of uncertainty, lack of control and the volatility of the obstetric environment [12]. One meta-analysis reports the prevalence of depression in fathers in the post-partum period to be 10% (25.6%; 95% CI, 17.3%-36.1%), noted to be highest in the 3-6 months after the birth [13]. Maternal depression, excessive infant crying, and maladjustment to parenthood also impact the mental health of fathers in the post-partum period [13–15]. The feelings of helplessness, fear and abandonment may precipitate anxiety, post-traumatic stress disorder (PTSD) and ‘sexual scarring’ [16–18]. Exposure to stressful circumstances such as a traumatic event in pregnancy and childbirth may also contribute to depression, acute stress disorder and PTSD in fathers [6, 16, 19–21]. Fathers of babies born preterm (< 30 weeks gestation) have significantly higher depression scores when compared to fathers whose babies are born at term [22]. A recent systematic review has highlighted that the impact of perceived stress on fathers in the perinatal period can contribute to mental health issues including anxiety, psychological distress, antenatal stress and depression [23].

Traumatic events during pregnancy are relatively common and are potential crucial contributors to men's mental health [24]. Fetal loss, including fetal death in utero, stillbirth and neonatal death, are unfortunate occurrences that are reasonably common in obstetric units [24]. In Australia, perinatal loss occurs in approximately 9.6 per 1,000 births, of which stillbirths (including antepartum and intrapartum fetal loss) comprise 7.1 per 1,000 births and neonatal deaths 2.5 per 1,000 live births [24]. Data on medically indicated termination of pregnancy for fetal anomaly is not readily available in Australia, but figures from the United Kingdom suggest that the age-standardised termination of pregnancy rate is 18.0 per 1,000 women [25]. The majority of these terminations (98%) are performed to protect the physical or mental health of the mother, while a small proportion (2%) are performed for fetal anomalies that may result in a substantial risk of serious physical or mental handicap in the offspring [25]. The psychological consequences of a fetal loss on mothers are well established; however, the impact on fathers is less well understood [5]. In the event of a termination of pregnancy, some fathers may struggle with the decision-making process due to doubt, guilt and a sense of failure [26].

Traumatic events can also occur during delivery and the postpartum period. Significant maternal bleeding at the time of childbirth, classified as a postpartum haemorrhage, occurs in 5-15% of births [27]. Additionally, 16% of liveborn babies require some form of active resuscitation immediately after birth [28]. Fathers witnessing these life-threatening situations may be left with long-standing post-traumatic stress symptoms (PTSS) [5, 17, 29, 30]. This trauma may culminate in guilt, self-blame, fear and shame, as well as perceived stigma [31–33]. Fathers are typically reluctant to seek help for mental anguish and use various coping strategies, including 'being strong', 'going back to work' and 'focusing on their partner' [12, 34]. This suggests that men may face significant mental health concerns when needing to support and care for their partner and baby, with societal obstacles in receiving health care for themselves. Men also have a significant impact on the future well-being of their offspring [35]. The children of depressed fathers have four times the risk of being spanked by age one and a less than 50% chance of being read to by their fathers [36]. Additionally, children of fathers with a significant psychiatric illness have double the risk of themselves being diagnosed with a psychiatric illness by age seven [37]. Paternal depression is similar to maternal depression with regards to impact on children’s internalizing and externalising behaviours [38]. Furthermore, adult children of depressed parents have an increased risk of major depressive illness and a five-fold increase in the risk of alcohol dependence [39]. Against this background, this study explores the emotional and behavioural responses and coping strategies of fathers or expectant fathers who faced a significant traumatic event during a partner's pregnancy.

## Methods

### Study design

A prospective qualitative study of male partners who experienced a traumatic event during the pregnancy, labour or postpartum period was undertaken. A “traumatic event” was defined as a medical incident resulting in serious risk to the mother and the unborn child, termination of pregnancy, intrauterine fetal death or stillbirth. The study was conducted at Redcliffe hospital, an outer-metropolitan public teaching hospital in Brisbane, Australia, which delivers approximately 1750 babies per year. Human research ethics approval was obtained and all methods utilized in the study were carried out in accordance with the relevant guidelines and regulations. The participants were recruited sequentially through the antenatal and ultrasound clinics and maternity ward by a senior obstetrician (AK) between September 2013 and March 2015. A total of 32 eligible fathers were approached, 28 were recruited, and 24 of these fathers agreed to be interviewed.

The interviews were conducted by a senior psychiatrist (GB) at 3-4 months after the traumatic event, using phone and face-to-face semi-structured interviews lasting from fifteen minutes to one hour and two minutes. The interview protocol has been included in supplement 1. Interviews were exploratory in nature and utilised broad questions and probing, resulting in detailed accounts of fathers' feelings and concerns. In the event of ambiguity, the interviewer asked further questions to clarify the intent and meaning of their response. If the subject matter caused distress to fathers, appropriate referral arrangements were made with the general practitioner or a specialist mental health professional.

#### Qualitative analysis

The interviews were professionally transcribed and analysed by all investigators using a preliminary automated process followed by a detailed manual qualitative analysis, performed using the six-phase guide proposed by Braun and Clarke [40, 41]. The qualitative approach used to interpret and understand the experiences and support needs of the fathers was underpinned by the social constructivist perspective [42, 43]. This outlook argues that people have different viewpoints about their social world, which, in turn, are influenced by their historical and cultural context. No pre-determined hypotheses guided the data collection.

The initial qualitative exploration was performed using a computer-assisted qualitative data analysis tool, Leximancer (Version 4.5, Leximancer Pty Ltd, Brisbane, Australia). In this automated analysis, semantic meaning is achieved through presence, frequency and co-occurrences of words and phrases leading to the generation of concepts, which are then clustered into higher-level themes [44, 45]. A stabilised visual concept cluster map of the qualitative data was generated, which demonstrated the relationships between the concept groups (bold labels) and dominant themes (coloured circles). The circles are named after the dominant concept within that group. Additionally, heat mapping with colour coding indicated the order of importance of the themes based on relevancies and connectivity score; ‘red and orange’ were given more emphasis and ‘blue and green’ showed less relevance and connection. The proximity of the concepts with each other also demonstrated how frequently they appeared in similar contexts. This automated analysis, while lacking the capacity to analyse implied meaning [46, 47] was used as a source of information for the detailed manual analysis that followed [40, 41].

During phase one of the manual analysis, the study investigators developed an immersive familiarity with the transcribed interviews and generated a list of initial ideas. Phase two utilised an inductive approach to generate codes, thus organising the data into meaningful groups. This step was carefully performed to ensure all data extracts were comprehensively coded and inclusive of their context before being finally collated. In phase three, different codes were sorted into potential themes and sub-themes. The data relevant to each theme across the entire dataset was actively identified and reviewed. Throughout phase four, the coded data extracts and themes were reviewed, refined and divided into individual groups that contained consistent information. In phase five, a latent level analysis was undertaken to look for repetitions, metaphors, analogies, as well as similarities and differences. The patterns and themes were combined, refined, separated or discarded and finally selected. A vertical analysis was performed to contextualise and semantically condense each source to develop an in-depth understanding of a particular theme. A horizontal analysis was then utilised to compare the results of the vertical analysis to generate an iterative interpretation of the entire dataset as a whole [48]. In phase six, the themes and sub-themes were reported and a thematic map was generated. Pertinent quotes from the narratives were chosen by re-reading and shortlisting the interview transcriptions for suitable illustrative examples.

#### Theoretical models that informed the final model

Thematic analysis was considered in the light of established theories. Detailed examination of personal lived experience, often results in complex, ambiguous and emotionally laden narratives [49]. It was clear that men’s background history, vulnerabilities and protective factors influenced their experience of the pregnancy and childbirth, so grounding this in theory was useful. A combination of two time-honoured theories, the Maslow’s hierarchy of needs and Calman’s quality of life gap theory, were chosen to develop the final thematic map [50, 51], as these theories were identified as best describing the emergent themes and constructs.

According to Manfred Max-Neef, fundamental human needs are finite, very few in number, classifiable and invariable [52]. These needs are context-specific and must be satisfied in relation to oneself, the social group and the environment. The Maslow’s five-tier model states that humans have physical, psychological, social and spiritual needs that provide meaning to life [50]. As a prerequisite, needs lower down must be satisfied to attend to needs higher up. However, recent interpretations suggest that needs may overlap or compete and can be influenced by cultural contexts [53]. After an extensive review of our data, we chose to represent our findings using an inverted and simplified version of Maslow's needs hierarchy pyramid.

The gap theory of quality of life, as proposed by Calman, was developed in cancer patients and explains the difference or gap between the hopes and expectations of the individual and their experiences [51]. A ‘gap’ is a theoretical construct subconsciously present in people’s minds reflecting the difference between expectation and reality. The greater the gap, the greater is the deviation from the theoretical ideal state of wellbeing. In terms of quality of life, individuals perceive their position in life in the context of multiple domains including culture, value systems, goals, expectations, standards and concerns [54]. Despite the hostility of the environment, individuals adapt by ‘making meaning’ and finding equipoise to achieve quality of life and a state of wellness [51].

Based on the analysis of the qualitative data, the theories of Maslow and Calman appeared complementary and synergistic with each other and most accurately symbolised the themes and meaning identified in our findings. A final explanatory model was constructed integrating the two theories together. It predicted men’s health following an adverse pregnancy event and provided insight into men’s adaptability and the role of health care support systems.

## Results

Twenty-four of the 28 participants (85%) who consented to participate in the study, completed the postpartum interviews; 3 declined and one was not contactable. The mean age of the participants was 32.9 years (standard deviation = 6.4). Most of the participants were Caucasian (89%), employed (77%) and had at least one previous child at the time of enrolment in the study (75%). Table 1 outlines the various categories of the maternal, fetal and neonatal causes of inclusion in the study. Fourteen fathers had maternal and fetal concerns, but took home a live baby, and 14 fathers experienced fetal loss.

**Table 1:** Reasons for inclusion in the study

**Maternal concerns (live baby)**	
Massive postpartum haemorrhage, maternal intensive care admission (2)	
Fetal structural anomaly, massive postpartum haemorrhage, maternal intensive care admission.	
**Fetal concerns (live baby)**	
Fetal structural anomaly (2), one with special care nursery admission	
Fetal structural anomaly (2)^a^	
Fetal genetic syndrome	
Neonatal intensive care admission	
Intrauterine growth restriction^a^	
**Maternal and fetal concerns (live baby)**	
Preterm delivery and maternal co-morbidities (2)	
Trisomy^b^, preterm delivery	
Trisomy^c^, preterm delivery	
**Termination of pregnancy**	
Fetal structural anomaly (4)	
Trisomy (4)	
Maternal co-morbidities	
**Fetal death/Stillbirth**	
Fetal death in utero (4)	
Stillbirth	

^a^Declined interview ^b^ Diagnosed at birth ^c^Declined termination

**Fig. 1:**
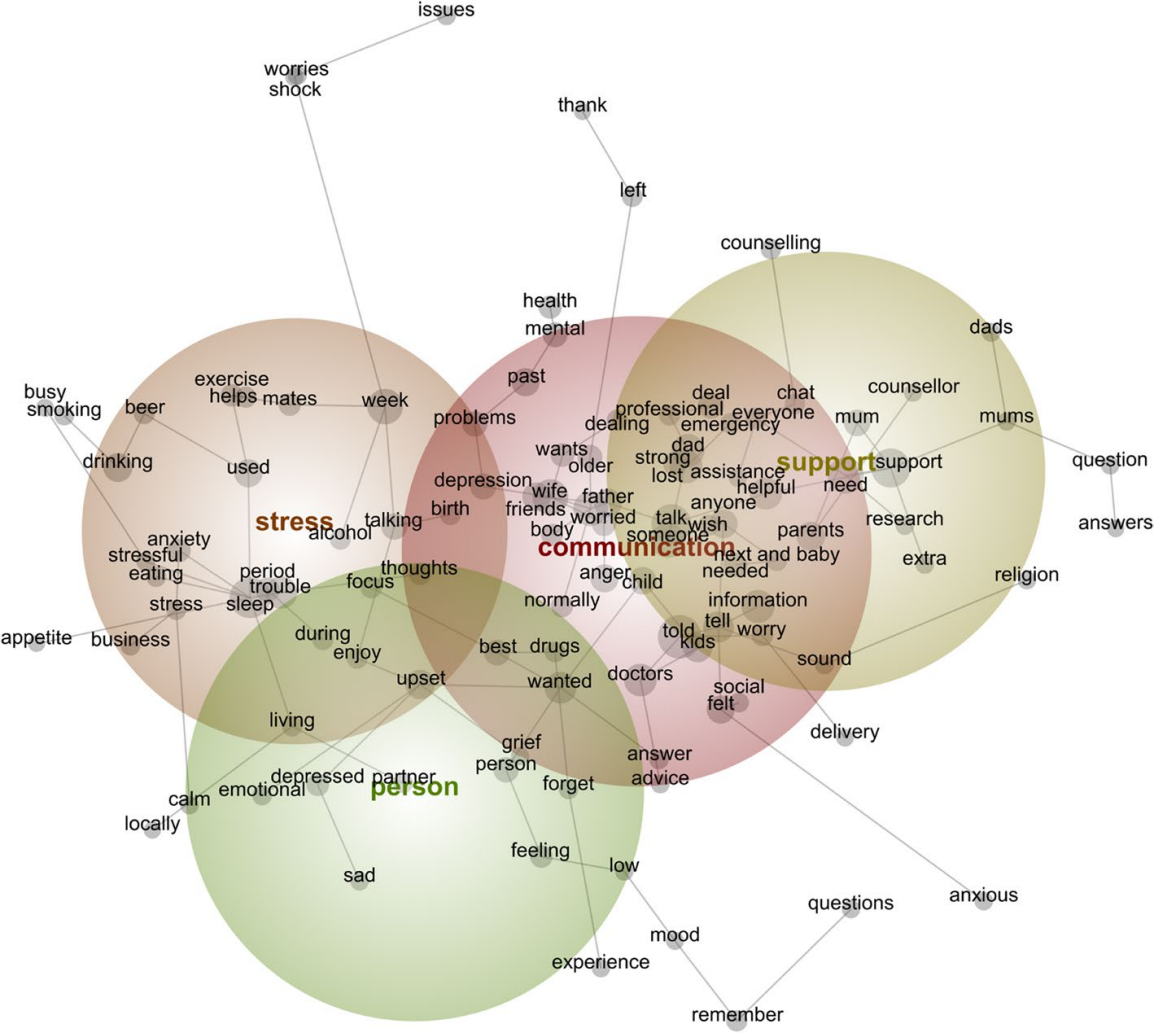
Leximancer generated concept map of the qualitative data

Figure 1 displays the themes identified in the automated analysis using Leximancer. The primary theme was ‘communication’ which was dominant to the other themes of ‘stress’, ‘supports’ and ‘person’. The three themes of ‘communication, stress and person’ were intricately linked with each other and could not be separated, whilst the theme of ‘support’ was only linked to ‘communication’.

**Fig. 2:**
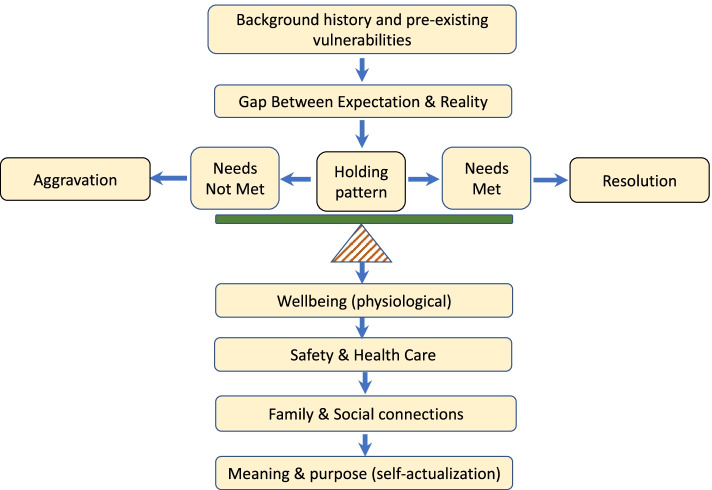
Final thematic map of the impact of an adverse pregnancy event on men’s needs

The main themes and concepts identified in the Leximancer analysis were also identified in the manual analysis. The dominant theme of ‘stress’ identified somatic symptoms such as sleep and eating concerns and adaptive and maladaptive coping strategies, such as exercise and drinking, respectively. Themes relating to somatic symptoms that were also identified in the manual analysis are presented in Table 3. The final thematic model arising from review of manual and automatic synthesis of the data is shown in Figure 2. The results of the manual analysis are described in further detail below with reference to the themes and sub-themes.

### Background history and pre-existing vulnerabilities

The reactions and coping strategies of the fathers were influenced by their previous life events and experiences. This included unpreparedness to deal with complications during childbirth, lack of parenting experience, previous loss of a loved family member, awareness of psychological supports, relationship stress, male stereotypes and spiritual growth contributing to resilience. Young, inexperienced and underprepared fathers described feeling unqualified and overwhelmed, especially when confronted with maternal complications or having to care for a newborn on their own. Some fathers had a previous history of physical and mental health problems, including complex trauma. One father admitted to a previous suicide attempt, and three others described losing their own father to self-harm. Exposure to difficult and trying circumstances in the past, predisposed some men to utilizing maladaptive coping strategies such as alcohol and drug abuse. Alternatively, a prior confronting life experience helped other men apply previously learnt adaptive coping strategies. Some men were already struggling with a vulnerable partner and a lack of family and social support systems. This resulted in further deterioration of circumstances, when fathers grappled with unresolved distress in their partner or difficult family interactions. However, strong relationships with a partner, family and friends, as well as a good rapport with a health professional, were identified by fathers as protective factors. Predisposing factors such as rigid personality traits also contributed to difficulties with coping. Many fathers acted within the constraints of male stereotypes by suppressing their true feelings and emotions (*n*=6). On the contrary*,* protective factors including religious beliefs, spiritual connections and their life perspectives promoted resilience resulting in post-traumatic growth. Some examples with illustrative quotes are presented in Table 2.

**Table 2:** Background and predisposing factors

Background vulnerabilities	
1. Lack of experience to deal with maternal complications: *“All these nurses started running around … I was in shock … my eyes were all watery … I couldn’t really see what was going on” (Participant 17).*	
2. Lack of parenting experience: *“I was pretty unprepared … I had never even held a baby before up until then, so I didn’t know what was going on”* (*Participant 17*)*.*	
3. History of loss: *"I was roundabout 14. My dad … he, committed suicide. It was just him and me living in the house at the time. I was the one who found him. It had a profound effect on the whole family”* (*Participant 17*)*.*	
4. Psychological awareness due to previous professional support: *“I had professional help in the past. That’s probably [why] I use some of my techniques or whatever they teach you when traumatic situation happens, [which] helps me to deal with stuff”* (*Participant 6*)*.*	
5. Relationship stress: *"Yeah, yeah not being rude, you know, at about the sixth week when I was coming home, I had an argument with her, and I said, ‘you got to try and get over it you know, I can't keep coming home and listening to this evening and night. I'd be better off going to the pub after work’"* (*Participant 16*)*.*	
6. Male stereotypes: *“I did feel left out for a little while there … No one really asked how you’re going, you know? Because you've sort of got to be the strong one because I suppose … when you see your wife that weak and vulnerable, there's no use the two of you being like that - but, yeah, there is [sic] times where you think - this happened to me as well. It's not just happen [ed] to her”* (*Participant 19).*	
7. Spiritual growth contributing to resilience: *“If stuff didn’t happen with [son’s name], I wouldn't be on the same path … I had a focus then … The experience with [son’s name] has helped me cope with all different things in life, made me stronger as a person. He's like my hero … I just think that it's given me the skills in life, especially coping in grief and that”* (*Participant 3*).	

**Fig. 3:**
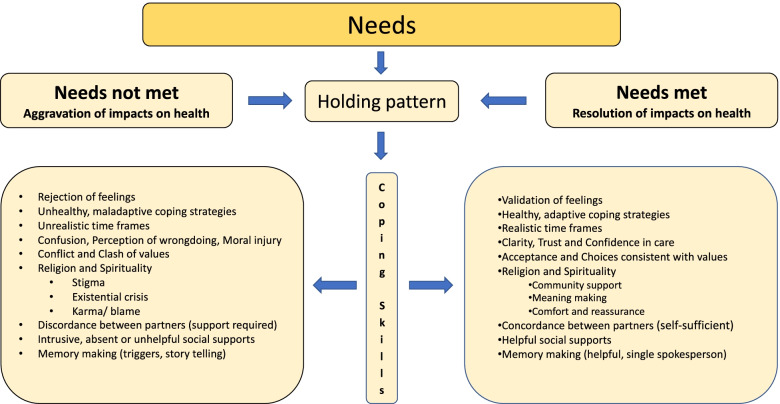
Participant needs and gaps (unmet needs)

The fathers in our study identified health as wellbeing across multiple domains, implying a ‘multidimensional positive state’. A significant traumatic event resulted in disruption of this complex adaptive system that maintained equipoise, resulting in an acute deterioration of wellbeing across emotional, spiritual and physical domains. The thematic model contains contrasting components in terms of responses relating to identified needs (Fig. 3). For example, some fathers had rigid expectations about the time frames required to get through the crisis, whilst others had a more flexible approach and seemed to cope better. Predisposing vulnerabilities such as a history of loss of a close family member or relationship vulnerabilities were associated with increased distress, whilst protective factors such as a prior positive experience with counselling was associated with an easier time in navigating this difficult period. Depending on their background history and pre-existing vulnerabilities as well as coping capabilities, most fathers would shift between their needs 'not being met' or 'being met', with a 'holding pattern' in between. However, this state was fluid and was described as changing from day to day.

### Gap between expectation and reality

The fathers expected the pregnancy and childbirth to be an ‘exciting and joyful experience’ for them and their family, resulting in a good outcome. However, any change to this circumstance would result in disappointment and a ‘gap between expectation and reality’. The participants “*felt lost, confused and empty”* due to the reality of their experience: *“It was mainly the roller coaster that led up to the whole end result. The cookie crumbled” (Participant 10).* When given news that his unborn baby had died, one father described how *"a happy day turned into complete chaos”* and all had been lost: *“It’s something that just hurts, but at the end of the day, the whole thing hurts"* (*Participant 5*)*.*

A father whose unborn child required multiple ultrasound scans at the hospital for a fetal condition felt the pregnancy was “*all-consuming*”: *“A time that should have been joy, lost its whimsy! We hated the pregnancy, to be honest with you... and you know everyone talks about this magical time, and we don't have it”* (*Participant 1*)*.*

In keeping with Maslow’s model, failure to have their needs met, adversely affected men’s health. The thematic map (Figure 2) depicts the needs in separate tiers, and each tier has been described separately and exemplified with illustrative quotes in the text.

### Tier 1. Physiological (mental and physical) wellbeing and coping strategies

In the event of an acute catastrophic event such as fetal death, several fathers suffered from acute anxiety, shock, helplessness, abandonment and terror (*n*=5). An uncertain diagnosis led to anger, disbelief, denial, resentment and reaction formation (*n*=7). Some fathers had obvious depressive symptoms (*n*=5), while others suppressed their grief (*n*=5). In the event of a fetal loss, some fathers struggled with anger (*n*=3), with the resultant grief leading to thoughts of self-harm (*n*=4). Several fathers lamented dashed hopes (*n*=5), felt helpless (*n*=3), overwhelmed (*n*=5) and suffered from flashbacks (*n*=5). One father felt unable to witness an induced birth of his baby that died in utero. Several fathers reported somatic symptoms such as poor sleep and appetite. Many felt dysfunctional and struggled to get through activities of day-to-day life. One father reported feeling so depressed that he was *“curled up on the lounge for about eight days”* and another “*lost six kilos in a week and a half from not eating.”* On the contrary, some fathers reported weight gain from stress eating and not getting the usual physical activity (*n*=9). One father admitted to behaving irrationally, and two reported paranoia and a feeling of impending doom (Table 3).

**Table 3:** Impact on physical and mental wellbeing of fathers

Physical and mental health impacts	
1. Abandonment: *"But at the time, thinking back now … anger, fear, anxiety, all that sort of stuff just started crashing down on the day … because my wife was still in surgery. I was with my son, and I got pushed outside, and all the curtains went around, so I didn't know what was going on"* (*Participant 6*)*.*	
2. Helplessness: *"It's very hard that the baby is dead and [we think] he is alive. He is moving, and she can feel the kicks, and I can feel the kicks. We [sic] knowing what could happen with the future of the baby"* (*Participant 1*)*.*	
3. Anger: *“I think it’s very important for fathers to get some support too, you know. When we were driving home from hospital, I was very angry, you know. That day, if someone spoke to me on the road, I probably would have done something bad”* (*Participant 15*)*.*	
4. Self-harm: *“Oh, I don't know, I always thought, what would happen if [partner’s name] or [baby’s name] didn't get through it? What would I do? That was sort of, well, would I do that, or would I not do that? That sort of suicidal thing. But it was sort of just, would I, wouldn't I?"* (*Participant 1*)*.*	
5. Feeling excluded: “*There was no time for them to talk to me or tell me what was going on. So, I was just standing in the corner while they were doing their job”* (*Participant 17*)*.*	
6. Frustration: *“But she couldn’t really go in a room by herself. She thought she saw [baby’s name] at one of the windows … [he] was haunting her. She was sticking to me like glue … because she didn’t want to be left alone. She just had a guilt thing; I think because we made that decision (to terminate), and she thought it was just karma coming back … she is from another country, no support here … I took a month off work"* (*Participant 15*)*.*	
7. Avoidance of distressing medical events: *“I wasn’t there for the delivery (of the stillbirth). She could have only felt it, but she didn’t see that stillbirth. I didn’t want to tell her, I didn’t want to be there, I didn’t want to have this image in my head”* (*Participant 18*)*.*	
8. Somatic symptoms: *“The whole period that she was in the hospital, I wasn’t eating or drinking or sleeping much at all … had severe anxiety for weeks”* (*Participant 3*)*.*	
9. Paranoid symptoms: *“I hear voices … one voice and then sometimes lots of voices. Sometimes I look back thinking someone calls my name, but no one there. Other people think I am freaky and tripping out”* (*Participant 7*)*.*	

Many fathers felt well supported by health professionals (*n*=10), whilst some reported feeling “*excluded*” (*n*=4). Three fathers felt frustrated by their partner’s reluctance to accept help from the care providers. These traumatic events were often followed by protracted periods where the fathers marshalled personal coping strategies. The effectiveness of these strategies was variable and included distractions such as exercise (*n*=7), computer gaming (*n*=2), solitude (*n*=1), alcohol (*n*=5) and drugs (*n*=1). Most of the fathers displaced their own needs by *“being strong*” for their partner (*n*=10) engaging with existing children (*n*=6) or by returning to work (*n*=6). One father went onto problem-solving and fixing mode to *“avoid feeling”* and *“getting on with it”* as a coping strategy. Several fathers coped by withdrawing and *“bottling up”* their emotions (*n*=6). One found “*going out fishing in a boat with mates”* helpful (*Participant 11*). Two fathers felt that “*time heals wounds,”* whilst others struggled to be present and listen, causing conflict. Occasionally, fathers coped with humour (*n*=1). Examples of the various coping strategies used by fathers are provided in Table 4.

**Table 4:** Coping strategies

1. Distraction: “*I am very lucky I am focusing hard on other things, that sort of takes up a lot of my mind space when the uncontrollable things happen. They can’t penetrate me fully”* (*Participant 20*)*.*	
2. Displacement of own needs: *“I mean obviously, I went straight back [to work]. I had two days off … I had to get on with life, get out, and deal with people sort of head-on … because you got to pay the bills, you got to work, and you got to come home, you got to deal with your family and, you know, you got to try to be strong”* (*Participant 15*)*.*	
3. Problem solving: “*Isn’t that what you guys do all the time? You know, this happens every week. What I wanted to know is what steps we needed to do next - this funeral and all these things”* (*Participant 20*)*.*	
4. Conflict: *“Definitely because me trying to give answers? I don’t know a lot of the answers … you just turn around, say you don't know, and things start [to] break down rapidly from that point" (Participant 10).*	
5. Humour: *"We just laugh at everything; what can you do? You can't get angry or frustrated"* (*Participant 26*)*.*	

### Tier 2. Safety and health care

Safety is a universal human need and men need to feel safe within the health care environment by having clarity and confidence in care [52]. They also require clear communication with health care professionals to entrust the care of their family members [6, 55, 56]. Although three fathers felt that the medical emergency had been handled well, many perceived the clinical care to be inadequate (*n*=6). Several fathers (*n*=10) felt frustrated due to problems with accessing medical information. Occasionally, a lack of visibility of the anomaly at termination led to confusion and mistrust of the health care professionals. Some fathers felt that they missed out on crucial conversations due to work commitments, and important information was lost in translation. Two fathers tried to gain support from online sources, although it was not perceived to be beneficial.

The most profound effects were noticed in the category of ‘fetal loss’. Many fathers, together with their partners, were faced with a difficult choice to proceed to a termination of pregnancy, when advised of a chromosomal abnormality (*n*=8). Some suffered from moral injury over the decision-making process, especially when it clashed with their own personal values. One father was resentful of the medical advice of a termination of pregnancy when his baby was diagnosed with a trisomy. He and his partner made the decision to continue with the pregnancy and subsequently delivered a liveborn son. Both parents chose not to have any further investigations performed, thus avoiding “*labelling him*”. Another father who had personal beliefs in the sanctity of life accepted the diagnosis of trisomy at birth (undiagnosed during pregnancy) and exhibited gratitude in the face of adversity. The diagnosis of a chromosomal anomaly was particularly traumatic for fathers when they were required to make the difficult decision with their partner to proceed with a termination of pregnancy or when faced with further adversity, such as premature delivery of the baby.

Several fathers grappled with the ‘unfairness of their life experience’. Even though the outcome was sub-optimal, some fathers maintained their faith and trust, potentially due to a good rapport with the health professionals and honest communication about the medical events. Other fathers appreciated by being ‘actively included’ in the conversations, exploring the impact of traumatic circumstances on the fathers (Table 5).

**Table 5:** Safety and health care

1. Perceived inadequacy of care: *“I was angry at; first, there was a little bit because of the negligence at the hospital. They hadn't done the procedure for about eight years, so there was where the baby complication has happened”* (*Participant 6*)*.*	
2. Clarity of care: *“Probably could have sat down with someone who could have explained to me step by step what was going on and what happened. All hospitals are a pretty rushy [sic] place … I couldn’t get a clear answer off any of them”* (*Participant 17*)*.*	
3. Confusion and mistrust: *“We had a hard time … we went into the hospital to have her induced … it normally only takes two days, but it took four days. When the baby came out, the head was perfectly normal … we were expecting something completely different, and we thought maybe they got it wrong, but the autopsy proved [that] there was something wrong"* (*Participant 19*)*.*	
4. Missing out on important information: *“And [partner’s name] she was there every day, and I have to come back from work. Looking at the baby sometimes when he cries, and we don't know what he is crying for. Looking at the machines and the oxygen level drops down and goes up again. Once we come back home- just tears”* (*Participant 22*)*.*	
5. Resentment over decision making: *“I was not even going to make the call and say yeah, go ahead with the termination. Because at the end of the day, if you make that call, because you'll never, ever know whether your baby has a chance. You've got to live with that for the rest of your life”* (*Participant 27*)*.*	
6. Support to accept difficult circumstances during pregnancy and childbirth: *"Yeah, she was six weeks early, and when she was born we were told … that she has Down syndrome. A bit overwhelmed by the way things went … but on the other hand, we got a beautiful little girl”* (*Participant 11*)*.*	
7. Support to grapple with the unfairness of life: “*It’s probably just the cards you have been dealt with … to tell you the truth...I am sort of a middle class, working guy. I don't smoke; I don't drink; I've never done drugs in my life. I [have] never done anything bad to anybody ever. It’s just like, pretty unfair to be dealt with”* (*Participant 10*)*.*	
8. Honest communication: “*So, it was annoying that we couldn't get an answer, but it's completely understandable, because why give an answer if you don't have it?”* (*Participant 1*)*.*	
9. Frustration with lack of explanation: *“Completely acceptable to me that sometimes these things happen. I wish there was a reason because my stupid practical mind would like a reason”* (*Participant 20*)*.*	
10. Inclusion in health care delivery: *“Because, you know, it is all about the mother and the baby. When the doctor came in … she wanted to know how it affected the father. I felt really good to be included”* (*Participant 11*)*.*	

### Tier 3. Connections-relationships, future pregnancy and support system

A shared traumatic pregnancy at times strengthened the relationship (*n*=4), by being *“better overall for the experience”* (*Participant 21*), and increased closeness with the partner. However, sometimes it contributed to new strains and discord within the relationship (*n*=3). Occasionally, it could be a real struggle for men ‘to be present and listen’ or there was a disconnect between both partners’ processing of events (*n*=3). Sometimes fathers struggled to negotiate a period where both partners had unmet emotional needs because of ‘being at different stages’ of managing their grief.

Contemplating a future pregnancy was often difficult among partners and frequently evoked feelings of anxiety, sometimes verging on dread. One father was particularly keen to have another baby, whilst others (*n*=4) were hesitant and fearful. One father reported sexual difficulties and remarked that ‘pressure to produce a pregnancy’ made him *“feel like a failure”*. Two fathers’ partners were already pregnant at the time of the interviews. One was quite ambivalent about the pregnancy and the other took on a spiritual perspective, seeing the new baby as almost a saviour in the terrible circumstances (Table 6).

**Table 6:** Impact on relationships and future pregnancy

**Relationships**	
1. Increased closeness: *“I think if [partner’s name] and I didn't have the relationship that we've always had over the 13 years, like a lesser relationship, I think it could almost end by these instances”* (*Participant 3*).	
2. New relationship strains: *“Yes, we had some pretty big arguments. And any little thing that I do, she would just take it out on me … Yeah, but it will end up making us stick together or make it stronger”* (*Participant 15*)*.*	
3. Unmet emotional needs: *"We put on a front. We don't let it out on each other or the kids, or other people. But I don't know what I want … she doesn't know what she wants”* (*Participant 5*)*.*	
4. Struggle to be present and listen: *“I didn’t really enjoy talking about it because it would just upset me a lot of the time. That’s where I probably let [partner’s name] down a lot … I totally shut down”* (*Participant 24*).	
5. Disconnect due to incongruent grieving: *“Yeah, we were just dealing with it - well, I think we were at different stages. Because through that whole thing, I was trying to be strong for her, and obviously, she's sick, and all she's worried about is getting better. By the time I got home, I was in that real deep emotional upset stage, whereas she was still focussing on her recovery”* (*Participant 3*).	
**Future pregnancy**	
1. Anxiety: *"You don't understand … if there is another nine months, which there has to be - that's going to be more like nine years! There won't be much sleep”* (*Participant 5*)*.*	
2. Fear: *“It’s hard to decide whether it would be exciting or terrifying”* (*Participant 10*)*.*	
3. Ambivalence: *“I wanted her to be fully ready and not just be rushing into this, just as a replacement for the one we lost”* (*Participant 24*)*.*	
4. Sexual difficulties: “*The other thing that's hurting a lot is, we have been trying to have another baby … So that's just added burden … Nothing works the way it used to … it's constant thoughts in my mind. If we are intimate - it scares me - what if it happens again? I don't get to where I need to be, if you know what I mean. I don't … I don't ever finish"* (*Participant 5*)*.*	
5. Spiritual perspective: *“If it wasn’t for [baby’s name], this baby would never be here. And all the things that baby achieved, again, would never happen if [baby’s name] were here”* (*Participant 20*)*.*	

Most fathers were aware of mental health supports (*n*=10). However, some fathers (*n*=2) were disappointed by the lack of support systems. Four fathers with substantive depressive symptoms accepted psychological help; however, most (*n*=7) declined the offer. One father felt that preferential support should rightfully be provided to the mother, while others (*n*=3) observed a lack of assistance for their partner.

Many of the participants reported on the importance of social connections with family (*n*=18), the presence of other children (*n*=6), male friends (*n*=3), and work colleagues (*n*=2). Some appreciated the accidental bonding with strangers over shared experiences (*n*=2). A father who endured fetal loss described the benefits of the family to help it *“get it off your chest, call some family members and just talk it out”* (*Participant 6*) and one described leaning on his father: *“he is my go-to guy when anything goes wrong”* (*Participant 1*)*.* Conversely, some participants who did not have a similar support network, lamented the lack of understanding by their friends and family. Well-meaning enquiries from relatives and acquaintances unaware of the traumatic events conjured unpleasant memories and one father felt that having a single spokesperson had a protective effect. Several fathers got emotionally triggered with repeated storytelling (*n*=4), whilst others [4] struggled with reminders of the lost child (Table 7).

**Table 7:** Support systems

**Mental health supports**	
1. Declined help: *“I would like to get into some sort of counselling. But where do you go? Who do you see? It's not the sort of done thing as a male”* (*Participant 5*).	
2. Lack of help for fathers: *“Well, not many people really asked … because everything was focused on my wife. I understand how you need more research on the fathers because, really, we've got to bite our tongue and try and stay strong. But in the end, I lost my son as well. So, it’s a lot of pain you’ve got to hide and not show. You break down when it happens. You’re both in a mess, but … there is that feeling where - it all focuses on the wife”* (*Participant 19*)*.*	
**Social supports**	
1. Social connections: “*Our next-door neighbour, 82, comes over and says she had a baby stillborn and never told anyone*” (*Participant 20*).	
2. Lack of understanding *“The family were pretty good at the start, but I don’t think they (really) understand what it is like to lose someone”* (*Participant 5*)*.*	
3. Triggers due to enquiries: *"I work in a pretty big company, and I'd just had two weeks off work. Every person I run into... sort of says, how's your holiday? Yeah, mate, it's not a holiday. Then after, probably the fourth or fifth day of being back, ready to punch anyone in the face who says, how's your holiday, mate?”* (*Participant 5*)*.*	
4. Repeated storytelling: “*It almost got to the point; we had to take the phone off the hook!”* (*Participant 18*).	
5. Emotional reminders: *“The hardest thing to deal with is … my sister [is pregnant]. They were a week after us. They've got their child being born … it was just a shame that ours didn't make it”* (*Participant 25*)*.*	

### Tier 4. Memory making and post-traumatic growth

Three fathers appreciated the opportunity to create mementos as tokens of remembrance of a lost baby. Some fathers had difficult conversations about the dead baby, wanting to forget and remember at the same time (*n*=2). Many fathers (*n*=11) found solace in transcendent philosophies of fate, faith, religion and spirituality. An indigenous father found traditional spiritual beliefs and totems comforting after his partner’s emergency hysterectomy. Another father found strength and meaning through Christian faith: *“God gave my son to us - so you just accept it”* (*Participant 26*)*.* However, faith could also be a double-edged sword as one father felt ‘ostracized’ by the church due to his decision to proceed with a termination.

Grief and a sense of loss were the primary experiences of many participants. Some fathers felt guilty (*n*=4) and blamed themselves (*n*=1) or others (*n*=1), although one displayed ‘comparative guilt’ by only caring for the sick mother and failing to connect with the sick newborn baby (both requiring emergency surgery).

Many fathers salvaged ‘meaning’ out of the difficult situation: *“Baby came on his own birthday and died on his own day”* (*Participant 19*)*.* One father even found ‘a sense of accomplishment in loss’ as it was a vaginal delivery (after three Caesareans)*.* A father who had a baby born with a genetic syndrome was keen to avoid any form of stigma by not disclosing the diagnosis even to close relatives. A father whose partner underwent a termination for a structural anomaly reframed the events in a spiritual perspective. Another father, whose partner suffered a sudden stillbirth, had concerns that the staff focussed on emotional support but not practical advice. This father and his partner sublimated their tragedy by raising funds to donate a “cold cuddle cot for stillborn babies” to the maternity unit (Participant 20) (Table 8).

**Table 8:** Mementoes, meaning and purpose

**Memory making**	
1.Constructive memory making: *“The only thing they said to us, is to make sure you get the photos, and looking back, I never looked at the photos because they took me back to the bad days … where everyone was crying. I know what had happened... Although we do have this little teddy bear that we gave to baby when she was born … we both kiss her teddy bear, like [our] baby is home with us"* (*Participant 20*).	
2. Spending time with stillborn baby: *"We got to see her every day for an hour or two … Just in coming to terms with her, I suppose … but I couldn't hold her at first. After a couple of days, I did finally hold her and looked at her, nothing wrong with her at all"* (*Participant 18*)*.*	
3. Reassuring indigenous beliefs: “*Whether it’s a bird on the tree … little signs that pop to let you know that everything will be ok”* (*Participant 3*).	
4. Faith: *“We were going to one church, but then we found out the pastor was very dead against us going there. Well, because he thought that we should not have gone through with the termination. He thought that we should have kept the baby and left the results up to God”* (*Participant 14*).	
5. Comparative guilt: *“I didn’t carry [baby’s name]. I didn’t know [baby’s name]. When everything came down, it might sound kind of bad, but I had no connection with her … All I cared about was [partner’s name]”* (*Participant 27*).	
**Meaning and purpose**	
1. Accomplishment in loss*: "The day of her birth, I was uncomfortably happy. I saw our baby girl, and it was the first baby that we had naturally. The others were caesareans, so it was an accomplishment … I can't explain it … but that day wasn't as bad as what I expected. Yes, there (were) emotions of pride … I didn't cry till the next day, and the next day I couldn't stop crying. But I don't cry, that's not what I do. I still feel guilty about the day she was born... I don't feel right that I was not (more) unhappy"* (*Participant 10*)*.*	
2. Avoiding stigma*: “We actually didn’t tell anyone. We haven’t told any family yet. I don’t think I am going to tell them, I just want them to treat him like a normal kid”* (*Participant 26*)*.*	
3. Reframing sad events: *"When we went through the termination, we met somebody … somebody older who said something … he said no one goes away - the spirit comes back”* (*Participant 2*)*.*	
4. Frustration with impractical advice: *“There was lots of people whom I didn’t know, telling me how sorry they were … and I thought ‘Come on, has it never happened before?’ I was always trying to cut through all the smoke to find these practical things. I actually said that to the doctor in charge - what is the next step?”* (*Participant 20*)*.*	

## Discussion

This prospective qualitative study of fathers in the setting of a traumatic pregnancy focuses on first-hand accounts of fathers’ lived experiences. The main themes and concepts identified in the automated qualitative evaluation were complimentary to the detailed manual qualitative analysis. A novel application of Maslow’s needs theory and Calman’s gap theory was utilised to conceptualise the universal requirements of men facing a traumatic pregnancy. This thematic model provided interpretative value in depicting the needs of fathers and perceived gaps in their care, within the setting of this research project. The two models worked synergistically and were scalable (Maslow) and generalisable (Calman) in mapping the themes in these specific circumstances. This research builds on the previous application of Maslow’s model in other populations such as counselling care of refugees, maternity needs of women in rural settings, self-actualisation in homeless men and adolescent depression and children in crisis [42, 53, 57–59].

### The impact of a traumatic pregnancy and childbirth

Fathers are encouraged to be involved in the birth process and are significantly affected when exposed to traumatic circumstances. Our study demonstrated that events like a life-threatening postpartum haemorrhage or unexpected resuscitation of the newborn may leave the fathers feeling *“lost”* and *“abandoned”,* as the health professionals focus on caring for the mother or the baby. There are no provisions for dedicated personnel to support the fathers in these challenging and unpredictable circumstances, even in the developed world. Such traumatic exposures may intensify their anxiety and distress [7, 12, 56] These traumatic experiences may also result in long term PTSS and PTSD, as previously reported in the literature [5, 6, 12, 17, 30, 60]

Participants in our study discussed ways in which a traumatic pregnancy and childbirth experience had given rise to feelings of guilt, anxiety, depression, possible PTSS and suicidal ideation, consistent with other studies in the literature [5, 6, 17, 30]. Currently, fathers are not routinely screened for physical or mental health conditions or other pre-existing vulnerabilities [61]. This is concerning as a previous mental illness can contribute to increased intensity of grief following a perinatal death [33].

The fathers echoed the sentiments of grief and guilt following a fetal loss, similar to the findings of a systematic review on the psychological effects of stillbirth and neonatal death on fathers [62]. The experience of a fetal loss culminates in guilt, self-blame, fear and shame, as well as social and religious stigma [31–33, 63]. Whether the decision is accepted or declined, a termination of pregnancy profoundly affects both parents [64–68]. This study provides an additional contribution to the inadequate literature on the impact of termination of pregnancy on fathers.

Our study reiterated that fathers might be reluctant to witness the stillbirth [69]. Furthermore, fathers may have different preferences regarding delivery of a dead baby. One father in our study preferred a vaginal delivery (after previous Caesareans), in contrast to another study where multiple fathers were triggered to request a Caesarean section [56]. Additionally, whilst some fathers may be grateful for the mementoes of their child, others may find this aspect distressing. They may however be appreciative of having ‘something to treasure’ at a later date [32, 56, 70]. Evidence-based guidelines on caring for families after a perinatal bereavement recommend empathetic, genuine and culturally appropriate care [71, 72].

### Stressors, coping strategies and psychological support systems

Our study demonstrated that most men struggled with communication, provision of medical information, difficult decision-making and relationship strains. Many fathers utilised active adaptive coping strategies, such as ‘being strong’, ‘finding meaning’, and even identifying a sense of achievement in loss. There were hints of defensive and mature strategies such as dissociation, reaction formation, displacement, intellectualisation, and sublimation and this area requires further exploration [73]. Some fathers used constructive coping such as exercise, counselling or religious advice and others engaged in maladaptive coping by engaging in substance abuse, as highlighted previously [5, 12]. While fathers with significant depressive symptoms were actively encouraged to accept mental health support, most declined help, keeping with previous literature [5, 12].

### Communication with partners, family and future pregnancy

Most of the participants in our study expressed gratitude to doctors and midwives, however, some fathers expressed dissatisfaction with health care communication, similar to other studies [6, 32]. Some fathers also highlighted inadequate supports for their partner after a fetal loss, as noted previously [32]. Emotional conflict due to incongruent grieving between partners was reported, sometimes leading to relationship failure, consistent with previous literature [74–76]. As observed in our study, men may have differing needs to their partner regarding a future pregnancy, and some may be terrified and become dysfunctional, whilst others may become anxious if conception is delayed [5, 77].

### Maternity services and the role of health care providers

Further research is required to quantify the impact of a traumatic pregnancy and childbirth and the complex support relationship of fathers with the mother. The social role of fathers as caregivers, supporters and protectors of their partners highlights their valuable involvement and support [62]. However, fathers reported being expected to care for their partners without considering their own feelings [78]. The degree to which coping style is adaptive or dysfunctional is individual, as it is influenced by background vulnerabilities and strengths, moulded by genetic and developmental factors. Every effort should be made to ensure that maternity services are ‘father inclusive’.

### Strengths and limitations

The strengths of this qualitative study include multiple perspectives from a relatively large participant cohort and excellent engagement with the researchers. This research was undertaken in a single institution and with a specific group of researchers and may not be generalisable to other populations or geographical locations. Although fathers from various ethnic backgrounds were coincidentally recruited, this study was not specifically aimed to study cultural differences. The participants comprised a heterogeneous group in terms of the traumatic event. This included maternal complications, stillbirth or fetal loss, while some fathers took home a baby that survived. This may be considered a limitation as well as a strength, as the themes were coherent across the dataset. The novel ‘needs and gap model’ provides an exceptional insight into how fathers may help themselves in extenuating circumstances and how health practitioners may support them to reach a state of resolution, acceptance, peace and return to function. This conceptual model was tested on fathers with similar experiences and appeared to fit their stated experiences. Further research is required to understand and evaluate the applicability of this thematic model in representative and diverse populations of fathers.

## Conclusion

The rich dataset in this study has allowed a unique insight into fathers' emotional and behavioural responses and coping strategies after stressful events in pregnancy and childbirth. These situations result in significant psychosocial consequences for fathers, with potential long-term consequences for themselves, their partners and babies. Paternal perinatal depression is a significant public health concern [13]. A multi-disciplinary approach to supporting fathers after traumatic events is urgently required. Further research should examine the vulnerability and protective factors identified in this study in a larger representative sample to direct future interventions. Additional research informing care at a national or global level in planning interventions for bridging these gaps will reduce risk and improve resilience (Table 9).

**Table 9:** Suggestions for future practice and health services implementation research

1. Utilise opportunistic contact with fathers to screen for pre-existing physical and mental health conditions.	
2. Father-focused antenatal education, including information on uncomplicated and complicated births.	
3. Increase awareness in fathers of mental health conditions and active encouragement to access support services.	
4. Cautious encouragement of fathers to hold the baby after fetal demise to aid memory creation due to the small, but definite risk of harm in some fathers.	
5. Greater exploration of the factors that impact fathers' mental health in the postpartum period, especially in the setting of a traumatic pregnancy.	
6. Create a safe environment for men, promoting positive emotion, social support, meaning-making and caring interaction with health care providers.	

## Supplementary Information

**Additional file 1.**

## Data Availability

Additional information for this manuscript is available from the corresponding author on reasonable request.

## Abbreviations

PTSS: Post-traumatic stress symptoms
PTSD: Post-traumatic stress disorder
