# Supplementary material for: “It’s a lot of pain you’ve got to hide”: a qualitative study of the journey of fathers facing traumatic pregnancy and childbirth
Source: BMC Pregnancy Childbirth. 2022 May 24;22:434. doi: 10.1186/s12884-022-04738-4 (PMC9128289; doi:10.1186/s12884-022-04738-4)
Supplement: Supplementary file 1 — Additional file 1. [file 12884_2022_4738_MOESM1_ESM.docx]

**INTERVIEW PLAN**

Thank you for agreeing to participate in our research.

This interview will be recorded for future study, and the recording will be stored securely and not used for anything other than the purposes of this study. Whilst it is our focus to assess your psychological health and wellbeing for purposes of research, if any need for psychological assistance becomes evident in our interview, we will advise appropriate support and coordinate this for you if requested and required.

Domains for assessment

1. **General demographics**- age, marital status, vocational status, children.
2. **General subjective assessment** of wellbeing since the pregnancy- if going well, brief assessment of mood, anxiety, neuro-vegetative function, then proceed to domain 4.

If not going well, proceed to domain 3.

1. **Extensive assessment of mood, anxiety and neuro-vegetative function** including, the pervasiveness of depressed mood, diurnal mood variation, feelings of hopelessness, guilty preoccupations, sleep disturbance, changes in appetite and weight, problems with fatigue, problems with concentration, presence of panic attacks and/or generalised anxiety, presence of post-traumatic stress symptoms including hyperarousal, re-experiencing phenomena and emotional numbing. Presence of suicidal ideation and or intent, presence of psychotic symptoms. Where appropriate, enquiry about other life stress source (especially marital strain), past mental health and medical history and substance use and any other relevant enquiries.
2. **Coping strategies-** how did the fathers deal with their current life circumstances, whether it be with a healthy baby and the general changes this brings, or with grief loss or the ongoing burden of a child with a serious disability? (the nature of the coping strategy is likely to be very variable depending on the circumstances).

- Do they cope using positive reinterpretation and growth and active coping-examples being ‘I try to grow as a person as a result of the experience’ is or ‘I look for good in something that has happened’ is or ‘I do what has to be done one step at a time’?
- Do they cope using acceptance ‘is I accept that this has happened and that can’t be changed’?
- Did they cope using religion? ‘I try to find comfort in my religion’.
- Do they cope using humour?
- Do They cope using mental or behavioural disengagement ‘do I sleep a lot more than I used to’, ‘I daydream a lot, I give up more easily than I used to.’
- Do they use denial?
- Is there a focus on venting and catharsis ‘as I let my feelings out’?
- Is working a useful outlet/coping strategy?
- Did they use exercise as an active coping strategy?
- Has there been increasing consumption of alcohol and drugs?

1. **Access to help**-if the fathers sought extra assistance, whom they turn to? (Family, friends, professional-informational, counselling, helplines).

Why did they choose a particular avenue of support?

If they saw professional help, were there barriers to access (not know where to look, difficulty accessing due to time restraints/work commitments, waiting times, stigma).

Was there more support available to their partner than to them?

Thank you very much for your assistance with our research.
